# A Systems Biology Approach to Understanding the Mechanisms of Action of Chinese Herbs for Treatment of Cardiovascular Disease

**DOI:** 10.3390/ijms131013501

**Published:** 2012-10-19

**Authors:** Bohui Li, Xue Xu, Xia Wang, Hua Yu, Xiuxiu Li, Weiyang Tao, Yonghua Wang, Ling Yang

**Affiliations:** 1 Center of Bioinformatics, Northwest A & F University, Yangling 712100, Shaanxi, China; E-Mails: 2009013706@nwsuaf.edu.cn (B.L.); xu_xue_1986@163.com (X.X.); fishery18@163.com (X.W.); yuhua200886@yahoo.com.cn (H.Y.); xiuxiuas123@163.com (X.L.); 1137074535@nwsuaf.edu.cn (W.T.); 2 College of Life Sciences, Northwest A & F University, Yangling 712100, Shaanxi, China; 3 Lab of Pharmaceutical Resource Discovery, Dalian Institute of Chemical Physics, Chinese Academy Sciences, Dalian, Liaoning 116023, China; E-Mail: yling@dicp.ac.cn

**Keywords:** systems pharmacology, ADME, Traditional Chinese Medicine, cardiovascular diseases

## Abstract

Traditional Chinese Medicine (TCM) involves a broad range of empirical testing and refinement and plays an important role in the health maintenance for people all over the world. However, due to the complexity of Chinese herbs, a full understanding of TCM’s action mechanisms is still unavailable despite plenty of successful applications of TCM in the treatment of various diseases, including especially cardiovascular diseases (CVD), one of the leading causes of death. Thus in the present work, by incorporating the chemical predictors, target predictors and network construction approaches, an integrated system of TCM has been constructed to systematically uncover the underlying action mechanisms of TCM. From three representative Chinese herbs, *i.e.*, *Ligusticum chuanxiong* Hort., *Dalbergia odorifera* T. Chen and *Corydalis yanhusuo* WT Wang which have been widely used in CVD treatment, by combinational use of drug absorption, distribution, metabolism and excretion (ADME) screening and network pharmacology techniques, we have generated 64 bioactive ingredients and identified 54 protein targets closely associated with CVD, of which 29 are common targets (52.7%) of the three herbs. The result provides new information on the efficiency of the Chinese herbs for the treatment of CVD and also explains one of the basic theories of TCM, *i.e*., “multiple herbal drugs can treat one disease”. The predicted potential targets were then mapped to target-disease and target-signal pathway connections, which revealed the relationships of the active ingredients with their potential targets, diseases and signal systems. This means that for the first time, the action mechanism of these three important Chinese herbs for the treatment of CVD is uncovered, by generating and identifying both their active ingredients and novel targets specifically related to CVD, which clarifies some of the common conceptions in TCM, and thus provides clues to modernize such specific herbal medicines.

## 1. Introduction

Cardiovascular disease (CVD) is an abnormal function of the heart or blood vessels. It can cause an increase in risk for heart attack, heart failure, sudden death, stroke and cardiac rhythm problems [1]. As the leading cause of death in the world, CVD has attracted unprecedented attention from medical researchers, and different types of drugs and medications are used in the treatment of this disease, such as intensive medication, revascularization therapy and traditional Chinese medicine (TCM) [2].

TCM, as a system of ancient medical practice that differs in substance, methodology and philosophy to modern medicine, plays an important role in health maintenance for the peoples of Asia, and is becoming more frequently used in countries in the West [3]. More than 15 million people in the U.S. consume herbal remedies, and the total number of visits to complementary and alternative medicine (CAM) providers far exceeds those to primary physicians, amounting for more than $34 billion out-of-pocket costs for CAM annually [4].

As one of the most widely used Chinese herbs for the treatment of CVD during the past decades, *Ligusticum chuanxiong* Hort. decreases the levels of serum cholesterol, lowers density lipoprotein, relieves the extent of atherosclerosis and reduces the red cell deformability *in vivo*, thus preventing atherosclerosis [5]. Also, *Ligusticum chuanxiong* can inhibit cell proliferation and protein synthesis, and increases nitric oxide (NO) production of rat vascular smooth muscle cells in a dose- and time-dependent manner. In addition, this Chinese herb appears to have a direct vasodilatation effect on isolated aortic rings induced by norepinephrine bitartrate (NE) and calcium chloride (CaCl_2_) [6]. *Dalbergia odorifera* T. Chen is regarded as a useful treatment for CVD and has been in widespread use in various Chinese herbal preparations, such as Danshen injection, Qi-Shen-Yi-Qi decoction, and Guan-xin-Dan-shen pills [7]. *Dalbergia odorifera* promotes the circulation of blood and relieves pain such as angina. It exhibits anti-inflammatory and antithrombotic activity that is of great importance for preventing the occurrence of chronic stable angina. Also, this herb has been used as an effective anti-platelet agent by preventing blood clot formation that can lead to heart attack or stroke [8]. As for *Corydalis yanhusuo* WT Wang, this Chinese herb is well known as a traditional Chinese herbal medicine used for CVD. The alkaloids extracted from *Corydalis yanhusuo* possess potent cardiovascular actions by significantly inhibiting the plasma endothelin-1 activity and preventing oxygen free radical injury and lipid peroxidation as evidenced by elevated superoxide dismutase (SOD) and glutathione peroxidase (GSH-Px) activity and a reduced MDA level [9]. This Chinese herb also reduces infarct size and improves heart function by inhibiting apoptotic cell death through modulation of the Bcl-2 family in myocardial I/R injury in rats [10]. In addition, *Corydalis yanhusuo* might act on opioid, γ-aminobutyric acid (GABA), or dopamine receptors, and thus alleviate painful conditions such as headache, chest pain, epigastric pain, abdominal pain and backache [11].

Although the therapeutic efficiency of the Chinese herbs *Ligusticum chuanxiong*, *Dalbergia odorifera* and *Corydalis yanhusuo* for the treatment of CVD has been evaluated and validated, several fundamental questions are still unanswered. What are the mechanisms of action of the three Chinese herbs? What are the precise targets of these herbs? What are the relationships between these herbals and diseases?

Unfortunately, because it is difficult to identify the potential targets and analyze the active substances of the herbs, the investigation of the above problems in molecular detail through experimental methods is still intractable. System biology, with its extremely high efficiency and molecular level representation, has been increasingly used in understanding the complex interactions of proteins and small molecules in a biological system and evaluating how these interactions give rise to the function and behavior of that system. It follows an interdisciplinary approach and combines the latest experimental methods in biology with knowledge and technologies in the fields of mathematics, computer science, physics and engineering.

Our previous work has firstly developed an integrated model of systems pharmacology to measure the efficacy of drugs, especially the multi-target drugs, and to reveal the functional mechanism of traditional medicine theories [12,13]. The model can be divided into three levels according to pharmacokinetics (absorption, distribution, metabolism and excretion, ADME): oral bioavailability prediction, multiple drug targets prediction and validation, network pharmacology techniques. Construction of the integrated model will be very helpful to explain the functions of herbal medicines and their relationships. Thus in this work, using the three representative Chinese herbs *Ligusticum chuanxiong*, *Dalbergia odorifera* and *Corydalis yanhusuo* for blood vessel treatment as the example, we firstly explored the potential targets of the three Chinese herbs, and then constructed their relationships with the relative diseases and signal pathways, which offers a great opportunity for the deep understanding of the biological basis of TCM.

## 2. Results and Discussion

Generally, drug discovery begins with the identification of a potential therapeutic drug target. The “target” is the naturally existing cellular or molecular structure involved in the pathology of interest that is specifically associated with the mechanism of action of drugs. In TCM, as one of the most important theories, “multiple herbal drugs for one disease” describes how different drugs treat the same disease, which implies the drugs probably share common targets. To validate this hypothesis, it is necessary to firstly identify the potential targets of the Chinese herbs, and then understand how the targets play roles in the disease process. Fortunately, recent advances in systems biology and medicine have allowed the application of new profiling technologies in the study of the potential targets of Chinese herbs. Successful prediction of the potential targets and understanding of the characteristic changes in molecular mechanism associated with “multiple herbal drugs for one disease” will facilitate the development of a novel disease diagnostic and stratification approach that will potentially lead to personalized healthcare strategies for a range of diseases.

### 2.1. OB Prediction and Analysis of Pharmacological Mechanisms

Many orally administered drugs must overcome several barriers before reaching their target sites. The first major obstacle to cross is the intestinal epithelium. This barrier function depends largely upon intracellular phase I and phase II metabolizing enzymes and specific membrane transport systems, including P-glycoprotein and MRP2 [14]. High oral bioavailability (OB) is often an important consideration for the development of bioactive molecules as therapeutic agents. Thus, an important goal for drug research is to gain sufficient understanding of the molecular properties that limit OB to facilitate the design of viable new drug candidates. Poor OB can result in variable exposure to active drug, especially for TCM considering that oral administration is the most desirable route of administration. In this section, we thus apply OB prescreening to determine whether a compound is pharmaceutically active in a TCM prescription. Table 1 shows that 64 of the 360 chemicals (17.8%) exhibit high OB.

#### 2.1.1. *Ligusticum chuanxiong* Hort

Twenty-two of 194 ingredients in *Ligusticum chuanxiong* demonstrate good bioavailability (60%) after oral administration. Interestingly, as the most abundant bioactive compound of *Chuanxiong*, Ligustilide (M120) only has an adequate OB of 50.10%, although it significantly inhibits the vasoconstrictions induced by norepinephrine bitartrate (NE) and calcium chloride (CaCl_2_) [6]. Indeed, this compound can be metabolized to butylidenephthalide, senkyunolide I (M156), and senkyunolide H (M155) *in vivo* [15]. The three natural ingredients produce various pharmacological activities in cerebral blood vessels, the general circulatory system and immune system including spasmolysis contraction effects [16,17], inhibitory effects of platelet aggregation [18] and anti-proliferative activity, and thus improve the therapeutic effect on patients. Cnidilide (M93, OB = 77.55%) and spathulenol (M169, OB = 82.37%) also closely correlate with the smooth muscle relaxant action, and thereby have the strongest spasmolytic activity [19]. Carotol (M8) and Ferulic acid (M105) with an OB of 149.03% and 86.56%, respectively, demonstrate better bioavailability compared with cnidilide and spathulenol, which show strong antifungal, antioxidant and anti-inflammatory activity [20,21]. The pharmacological activity of ferulic acid results in the improvement of blood fluidity and the inhibition of platelet aggregation [22], which may offer beneficial effects against cancer, CVD, diabetes and Alzheimer’s disease [23]. As for 3-*n*-butylphthalide (M85, OB = 71.28%), this compound is not only able to inhibit platelet aggregation, but also decreases the brain infarct volume and enhances microcirculation, thus benefiting patients with ischemic stroke. The above results reveal the main pharmacological effects of *Ligusticum chumanxiong* Hort., *i.e.*, significant antispasmodic and antiplatelet effects.

In fact, platelet aggregation represents a multistep adhesion process involving distinct receptors and adhesive ligands, with the contribution of individual receptor-ligand interactions to the aggregation process depending on the prevailing blood flow conditions, implying that the rheological (blood flow) conditions are an important impact factor for platelet aggregation [24]. Moreover, thrombosis, the pathological formation of platelet aggregates and one of the biggest risk factors for CVD, occludes blood flow causing stroke and heart attack. This explains why the traditional Chinese herb *Ligusticum chuanxiong* that disperses blood stasis, activates blood and promotes blood circulation has the antiplatelet activity, and thereby underlines why such a herb can be used for the treatment of CVD.

#### 2.1.2. *Dalbergia odorifera* T. Chen

Twenty-six percent (24 of 93) of the ingredients in *Dalbergia odorifera* meet the OB > 60% criterion irrespective of the pharmacological activity. Relatively high bioavailability values were predicted for the mainly basic compounds odoriflavene (M275, OB = 84.49%), dalbergin (M247, OB = 78.57%), sativanone (M281, OB = 73.01%), liquiritigenin (M262, OB = 67.19%), isoliquiritigenin (M259, OB = 61.38%) and butein (M241, OB = 78.38%). Interestingly, all of the six ingredients show obvious anti-inflammatory property. Butein, liquiritigenin and isoliquiritigenin inhibit cell inflammatory responses by suppressing the NF-κB activation induced by various inflammatory agents and carcinogens, and by decreasing the NF-κB reporter activity induced by TNFR1, TRADD, TRAF2, NIK, TAK1/TAB1, and IKK-beta [25,26]. Besides the anti-inflammatory activity, odoriflavene has antioxidant effects and can significantly inhibit prostaglandin biosynthesis, as well as platelet aggregation [27].

Inflammation, which occurs as a response to cancer, has two stages, acute and chronic. Acute inflammation, the initial stage of inflammation, represents innate immunity and lasts for a short period and generally is regarded as therapeutic inflammation. If the inflammation persists for a long period of time, however, the chronic inflammation has been linked with most chronic illnesses, such as cancer, CVD and diabetes [28,29]. Obviously, inflammation occurs as a response to CVD, and *Dalbergia odorifera*, one of the most potent anti-cardiovascular and anti-cerebrovascular agents, exerts great anti-inflammatory activity.

#### 2.1.3. *Corydalis yanhusuo* WT Wang

*Corydalis yanhusuo* has gained ever-increasing popularity in today’s world because of its therapeutic effects for the treatment of cardiac arrhythmia disease, gastric and duodenal ulcer and menorrhalgia [30]. In our work, 21% (15 of 73) of chemicals in this Chinese herb display good OB (60% or even high), and the four main effective ingredients are natural alkaloid agents, including medicarpin (M263), dehydrocorydaline (M318), tetrahydropalmatine (M356), and dehydrocavidine (M316). Medicarpin and tetrahydropalmatine with an OB of 76.02% and 75.78%, respectively, enable the inhibition of the occurrence of inflammatory disease while also exhibiting potent anti-fungal activity by inhibiting germination and the hyphal growth of spores [31,32]. In addition to the anti-inflammatory anti-ulcer activities, dehydrocorydaline blocks the release of noradrenaline from the adrenergic nerve terminals in both the *Taenia caecum* and pulmonary artery, and thereby inhibits the relaxation or contraction of adrenergic neurons and relieves pain [33]. As for dehydrocavidine with an OB of 47.59%, this alkaloid exhibits a significant spasmolytic effect, which acts via relaxing smooth muscle, and displays inhibitory activity against HBsAg and HBeAg [34]. For the *Corydalis yanhusuo*, this Chinese herb exhibits a great effect in the treatment of inflammation and inhibition of pain. Evidently, these results provide concrete evidence for the efficiency of *Corydalis yanhusuo* for the treatment of CVD.

### 2.2. Target Identification and Validation

In recent years, CVD has been at the top list of the most serious health problems. Although many different types of therapeutic targets have already been identified for the management and prevention of CVD, such as endothelin, urotensin-II, low-density lipoprotein [35–37], lots of fundamental problems concerning CVD treatment with TCMs are, however, still unclear. For example, what are the interactions of the active ingredients of the Chinese herbs with their protein targets in a systematic manner? How do the corresponding targets change under differential perturbation of the chemicals? In this section, on the basis of the Random Forest (RF) and Support Vector Machine (SVM) methods, we have developed a robust and unbiased approach to probe the proteins that bind to the small molecules of interest in CVD. It combines the chemical, genomic and pharmacological information for drug targeting and discovery on a large scale [13].

Applied to 64 ingredients derived from the three traditional Chinese medicines *Dalbergia odorifera*, *Ligusticum chuanxiong* and *Corydalis yanhusuo*, which show good OB, 261 ligand-target interactions have been constructed, 221 of which are enzymes, receptors, and ion channels. This indicates that chemicals with multiple relative targets are responsible for the high interconnectedness of the ligand-target interactions. Indeed, it is the promiscuity of drugs that restrains the advance in recent TCM, because they were thought to be undesirable in favor of more target-specific drugs.

To validate the reliability of these target proteins, we have performed a docking analysis to select the ligand-protein interactions with a binding free energies of ≤−5.0 kcal/mol, which leads to the sharp reduction of the interaction number from 5982 to 760. These drug target candidates were subsequently subject to PharmGkb (available online: http://www.pharmgkb.org; accessed on 1 December 2011), a comprehensive disease-target database, to investigate whether they were related to CVD or not, and finally, 54 proteins were collected and retained (Table S2). Fourty-two proteins (76%) were identified as the targets of *Ligusticum chuanxiong*, such as dihydrofolate reductase (P150), an androgen receptor (P210) and angiotensin-converting enzyme (P209) that were involved in the development of CVD [38–40]. Of the proteins, seven and two were recognized as those of *Dalbergia odorifera* and *Corydalis yanhusuo*, respectively. For *Dalbergia odorifera*, this Chinese herb has 48 potential protein targets, 13 of which have at least one link to other drugs. The three herbs share 29 common targets, accounting for 52.7% of the total number. Indeed, as one of the most important doctrines of TCM abstracted from direct experience and perception, “multiple herbal drugs for one disease” has played an undeniable role. With the rapid advance in modern science and technology, people have recognized microscopic changes in human organs at cell and molecular levels. However, the potential correlation between TCM theories and microscopic changes has rarely been studied [41]. In this section, we have explored the potential targets of the three Chinese herbs, indicating that these drugs enable to target the same targets simultaneously and thereby exhibit similar pharmacological effects on CVD, which explains the theory of “multiple herbal drugs for one disease”.

Interestingly, we have also found that the three Chinese herbs possess specific targets. For example, the Chinese herb *Ligusticum chuanxiong* identifies the protein caspase-3 (P184), a cysteinyl aspartate-specific protease, as one of its specific targets, and exhibits inhibitory effects on the activity of this protease. In fact, connective tissue growth factor enables the activation of caspase-3 to induce apoptosis in human aortic vascular smooth muscle cells. Thus, modulation of the activity of caspase-3 with *Ligusticum chuanxiong* suggests an efficient therapeutic approach to CVD [42]. The Chinese herb *Dalbergia odorifera* has the α-2A adrenergic receptor (P216) as its specific target and probably blocks the release of this receptor, and thus influences its action [43]. A previous study has reported that the α-2A adrenergic receptor could regulate the release of norepinephrine from cardiac sympathetic nerves that were implicated in the development and progression of heart failure [44]. This indicates the efficiency of *Dalbergia odorifera* in decreasing the risk of heart failure. As for *Corydalis yanhusuo*, the protein tyrosine-protein kinase JAK2 (P9) is the only specific target of this Chinese herb. These results reveal the difference in the specific targets possessed by the three Chinese herbs, which finally induce the pharmacological disparities of the three herbs as mentioned in Section 3.1.

### 2.3. Network Construction and Analysis

The therapeutic efficacy of a TCM depends on multiple components, targets and pathways. The complexity becomes a huge obstacle for the development and innovation of TCM. To reveal the functional mechanisms will be the first step to solve this problem. With the development of pharmacology, the techniques of network pharmacology were considered as a potential tool for life science. Pharmacological network is a sort of a complex network that is used to reveal the relationships of Chinese herbal medicines with relative diseases and signal pathways. It is composed of nodes and edges. Such a network can be divided into four levels according to the different types of nodes and edges: ligand-candidate target network, ligand-potential network, target-disease network and target-pathway network. The nodes in the modular network present the units of function and the edges indicate the transmission and restriction between nodes. Construction of a functional network will be very helpful in explaining the functional modules and their relationships. In this section, using the three cardiovascular TCM *Dalbergia odorifera*, *Ligusticum chuanxiong*, *Corydalis yanhusuo* as examples, the target-disease and target-pathway connections have been constructed to investigate the pharmacological mechanisms of herbs related to CVD.

#### 2.3.1. Ligand-Candidate Target and Ligand-Potential Target Networks

**Figure 1:**
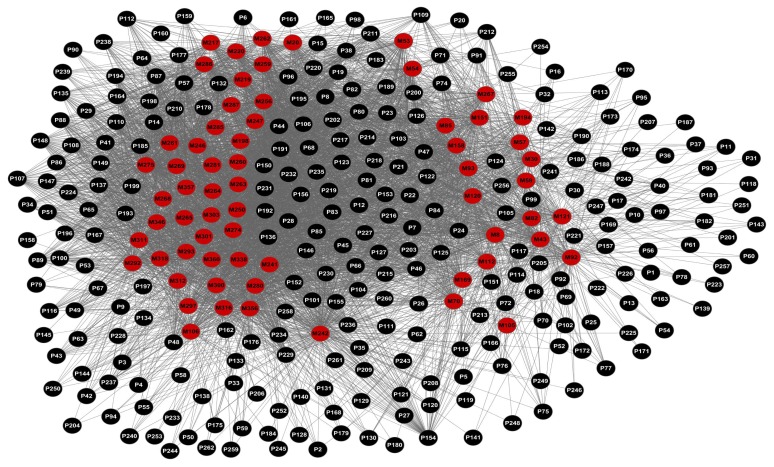
Network of 64 ligands (M) predicted to have 261 protein targets (P). The red nodes represent the ligands, while the black ones represent the proteins.

To further clarify the relationships between the ingredients of the Chinese herbs and their relative targets, we firstly constructed the ligand-candidate target network by connecting 64 chemicals and 261 protein targets. As shown in Figure 1, the network consists of 325 nodes and 5982 edges. For most chemicals, they are only linked to one or two targets, while some have more than three targets. Molecule 242 (butin) exhibits the highest number of target connections (173), following is Molecule 316 (dehydrocavidine) with 142 targets, and Molecule 194 (vanillic acid) has the least targets (38). Previous studies have already reported the relationships of the small molecules with CVD, which indicates the reliability of our results [45,46]. Regarding the candidate targets, we have found that prostaglandin G/H synthase 2 (P46) and prostaglandin G/H synthase 1 (P47) possess the largest number of connected ingredients (63). Following are nitric-oxide synthase, endothelial (P66) and tyrosine-protein phosphatase non-receptor type 1 (P8), which have 62 and 61 linked chemicals, respectively.

**Figure 2:**
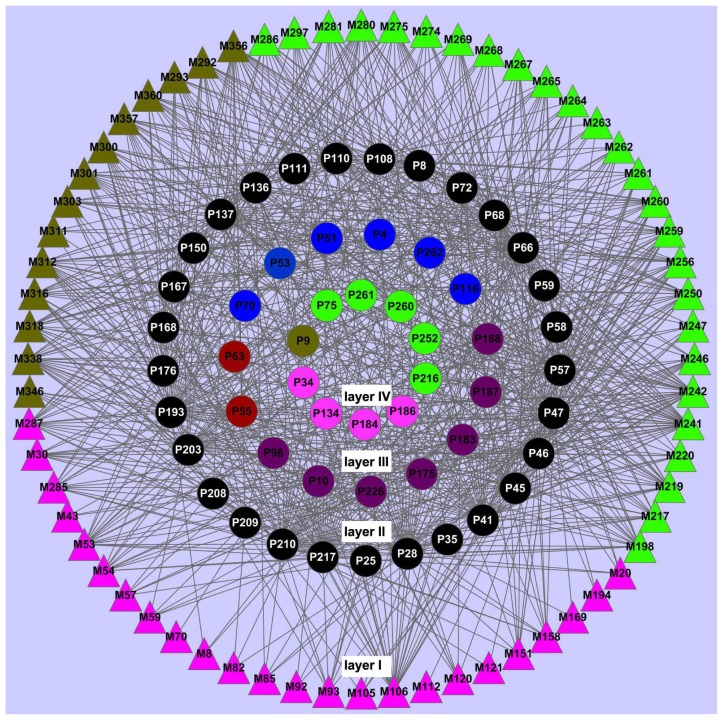
Network of 64 ligands (M, triangle, Layer I) predicted to have 54 protein targets (P, circle) after molecular docking validation. The black circles (Layer II) are the common targets of the three Chinese herbs *Ligusticum chuanxiong, Dalbergia odorifera* and *Corydalis yanhusuo*; the purple circles (Layer III) are the common targets of *Ligusticum chuanxiong* and *Dalbergia odorifera*; the red circles (Layer III) are the common targets of *Ligusticum chuanxiong* and *Corydalis yanhusuo*; and the blue circles (Layer III) are the common targets of *Dalbergia odorifera* and *Corydalis yanhusuo*. P9 (Layer IV) represent the protein target that is only identified for *Corydalis yanhusuo* (brown triangle); P34, P134, P184 and P186 (Layer IV) are only recognized by *Ligusticum chuanxiong* (pink triangle); and P75, P216, P252, P260, P261 (Layer IV) are only involved in *Dalbergia odorifera* (green triangle).

After the validation of molecular docking analysis, we then generated the ligand-potential target network derived from the ligand-candidate target network (Figure 2). This network comprises 118 nodes and 760 edges, with 64 chemicals and 54 potential targets. Of all the 64 ingredients, 39 have a relatively strong interaction with ≥10 potential targets, and 30 compounds are linked to more than 13 targets. Compound 241 (butein) exhibits the highest number of interactions with 31 protein targets, following are Compound 242 (butin, 26 targets), Compound 356 (tetrahydropalmatine) and Compound 259 (isoliquiritigenin) with 22 targets. Likewise, we have also found that many potential targets can be identified by more than one ingredient of the Chinese herbs. For example, nitric-oxide synthase (P66), prostaglandin G/H synthase 1 (P47) and cAMP-specific 3′,5′-cyclic phosphodiesterase 4A (P193) are examples of highly connected targets, the numbers of chemicals of which are 50, 48 and 45, respectively. Indeed, previous work has already confirmed the association between the protein targets and CVD, which indicates the reliability of our results [47–49]. This indicates that multiple proteins closely associated with CVD might share similar binding patterns with the ingredients of the Chinese herbs. The targets that are specifically identified by the three Chinese herbs, such as caspase-3 for *Ligusticum chuanxiong*, α-2A adrenergic receptor for *Dalbergia odorifera* and tyrosine-protein kinase JAK2 for *Corydalis yanhusuo*, probably possess different binding properties with the active substances of Chinese herbs, as mentioned in Section 3.2.

#### 2.3.2. Target-Disease Network

**Figure 3:**
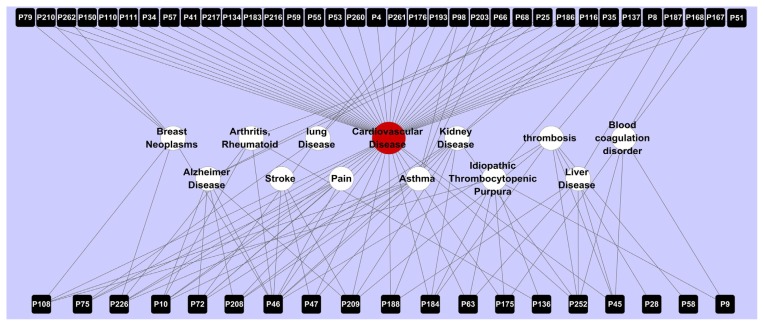
Network of 54 targets (P, black) connected to cardiovascular disease (red) and 12 other diseases (blank).

As shown in Figure 3, 54 target proteins (nodes) are connected to CVD (edges), 35 of which have at least one link to other diseases, such as pain, stroke, kidney disease, and Alzheimer disease. This explains why one Chinese herb can constitute a formula with different herbs for the treatment of various diseases to some extent.

The 29 targets shared by the three traditional Chinese herbs exhibit a high degree of correlations with CVD, which further verifies their effectiveness for treating this particular disease. For example, the heat shock protein HSP 90-alpha (hsp90-α, P108), highly expressed in the atherosclerotic lesions of humans, is the common target of *Dalbergia odorifera*, *Ligusticum chuanxiong*, *Corydalis yanhusuo*. This protein can stimulate the activation of the extracellular signal–regulated kinases (ERK), and thereby initiate an innate immune response, including the production of proinflammatory cytokines by macrophages and adhesion molecules in endothelial cells, which implies the role of hsp90-α in serving as a mediator/inducer of atherosclerosis [50]. Interestingly, this target protein is also connected to pain, a primary reason people seek medical care. This protein enables the activation of the signaling of spinal cord microglial toll-like receptor 4 (TLR4), and thus enhances neuropathic pain, which verifies the reliability of our target-disease network, and also implies the possibility of the above three herbs as pain killers. For the common target neuronal acetylcholine receptor subunit alpha-7 (nAChR-α-7, P72), this ion channel significantly inhibits the release of macrophage TNF, and attenuates systemic inflammatory responses, which thus reduces morbidity in CVD. Also predicted as a target protein correlated with pain, this protein is found to suppress mechanical pain responses associated with peripheral neuropathy and to accelerate functional recovery of the injured neurons, which has implications for the potential development of *Dalbergia odorifera*, *Ligusticum chuanxiong*, *Corydalis yanhusuo* for the treatment of pain.

These results provide a clear view of the relationships of the target proteins with CVD and other related diseases, which actually link the Chinese herbs and the diseases via the protein targets. This result confirms the efficiency of the herbs for the treatment of CVD and further explains the theory of “multiple herbal drugs for one disease” based on molecular pharmacology.

#### 2.3.3. Target-Pathway Network

Cells communicate with each other using a “language” of chemical signals. The cell grows, divides, or dies according to the signals it receives. Signals are generally transferred from the outside of the cell, through the cytoplasm and into the cell nucleus. Specialized proteins are used to pass the signal—a process known as signal transduction. Cells have a number of overlapping pathways to transmit signals to multiple targets. Ligand binding in many of the signaling proteins in the pathway can change the cellular communication and finally affect cell growth and proliferation. Evidently, studying the relationships between the target proteins and the related signal pathways, *i.e.*, constructing the target-pathway network related to CVD will provide more information on the mechanisms of action of Chinese herbs and potential side effects.

**Figure 4:**
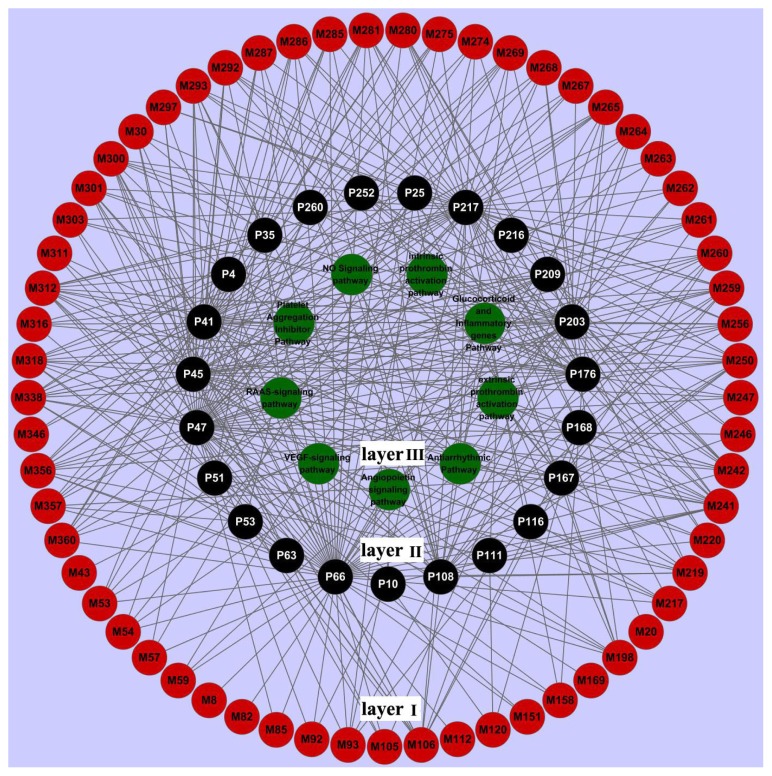
Network of nine signal pathways (green, layer 3) connected to 23 targets (P, black, layer 2). The ligands (M, red, layer 1) are linked to the targets.

We have extracted nine signal pathways closely associated with CVD in PharmGkb (available online: http://www.pharmgkb.org; accessed on 1 December 2011), including the renin-angiotensin-aldosterone (RAAS) pathway, the vascular endothelial growth factor (VEGF) pathway, the angiopoietin pathway, the antiarrhythmic pathway, the extrinsic prothrombin activation pathway, the glucocorticoid and inflammatory genes pathway, the intrinsic prothrombin activation pathway, and the platelet aggregation inhibitor pathway. As shown in Figure 4, 23 protein targets are linked to one or more signal systems.

RAAS is a hormone system that regulates blood volume and systemic vascular resistance, which together influences cardiac output and arterial pressure in the development of CVD [51]. When blood volume is low, cells in the kidneys secrete renin (P35) directly into the circulation. This proteolytic enzyme stimulates the formation of angiotensin I in blood and tissues. The inactive decapeptide angiotensin I is subsequently converted to the octapeptid angiotensin II by angiotensin converting enzyme (ACE, P209) in the lungs. ACE is a membrane-bound exopeptidase on the plasma membranes of cells and metabolizes a number of other peptides, including the vasodilator peptides bradykinin and kallidin, to inactive metabolites [52]. Finally, angiotensin II inhibits neuronal nitric oxide synthase (eNOS, P66) activation through the ERK1/2-RSK signaling pathway [47], regulating fluid balance and the secretion of aldosterone. Evidently, renin, angiotensin-converting enzyme, and eNOS are three important components of the RAAS signal pathway, and therefore they are the common targets of the three traditional Chinese herbs *Dalbergia odorifera*, *Ligusticum chuanxiong*, *Corydalis yanhusuo*. This result indicates that these herbs exert potential pharmacological effects on the RAAS systems via the three target proteins, which are associated with CVD.

Angiogenesis is a process that is of critical importance to tumorigenesis and tumor metastasis, as well as to the growth and maintenance of normal vasculature. In the VEGF signaling pathway, VEGF is an angiogenic factor that is very closely associated with the induction and maintenance of neovasculature in human cancers [52]. The receptors tyrosine kinases KDR/Flk-1 and Flt-1 are the VEGF receptors and play important roles in transducing signals upon VEGF stimulation of the endothelium. After the binding of the activated VEGF receptors the proto-oncogene tyrosine-protein kinase Src (P41) is activated in endothelial cells, which finally participates in the modulation of VEGF-dependent vascular permeability. Also, VEGF is found to stimulate the expression of eNOS and hence results in a maintained increase in NO formation, which is important in the process of VEGF-induced angiogenesis [53]. The protein hsp90-α interacts with eNOS, thus serving as a molecular scaffold to promote phosphorylation of eNOS and VEGF-stimulated NO production [54]. As the main components in the VEGF system, proto-oncogene tyrosine-protein kinase Src, eNOS, and hsp90-α is also recognized as common targets of *Dalbergia odorifera*, *Ligusticum chuanxiong* and *Corydalis yanhusuo*, which are efficient for the treatment of CVD. This implies that the candidate drugs can target different target proteins involved in the same or different signal pathways, and thereby have potential effects on the whole signal system.

## 3 Materials and Method

### 3.1. Dataset Construction

All ingredients of the three herbal medicines were extracted from our own database: TcmSP: Traditional Chinese Medicines for Systems Pharmacology Database and Analysis Platform [55] including 194 in *Ligusticum chuanxiong*, 93 in *Dalbergia odorifera* and 73 in *Corydalis yanhusuo*. All these chemicals were saved as mol2 format for further analysis. The information about the compounds is shown in Table S1 or can be obtained directly from the online database [55].

### 3.2. Oral Bioavailability Prediction

Oral bioavailability (OB) is an important pharmacokinetic parameter among ADME properties in drug screening cascades and represents the percentage of an orally administered dose of unchanged drug that reaches the systemic circulation. In our previous work, we developed a robust *in silico* model integrated with the metabolism (P450 3A4) and transport (P-glycoprotein) information to predict a compound’s oral bioavailability, which was supported by a dataset of 805 structurally diverse drug and drug-like molecules [56]. This powerful prediction model eliminated the influence of complex adsorption and metabolic processes, and was considered for two purposes: (1) dataset division; (2) probing binding features of chemicals.

The set of the drug compounds we retrieved was firstly divided into four subsets based on P450 3A4 docking score. Then, the DRAGON professional (version 5.6; Talete SRL: Milano, Italian, 2006) was applied to calculate the descriptors of each subset. The optimal OB predicting model using Support Vector Machine (SVM) exhibits a high predictability (The regression coefficients are *R*_training_ = 0.89 and *R*_test_ = 0.85 for the internal training and external test data, respectively, with the standard errors of estimate SEE_training_ = 0.35, SEE_test_ = 0.42, respectively). With this powerful prediction model, the big and complex database was simplified to a small subset, and the compounds with good OB were screened out. The compounds were selected as candidate compounds according to the level of OB (≥60%).

### 3.3. Target Prediction

In search of the candidate targets, we have recently developed a systematic model that efficiently integrates the chemical, genomic and pharmacological information for drug targeting and discovery on a large scale, and is based on the two powerful methods Random Forest (RF) and Support Vector Machine (SVM) [13]. This model is supported by a large pharmacological database of 6511 drugs and 3999 targets extracted from the DrugBank database (available online: http://drugbank.ca/; accessed on 1 June 2011), and shows an impressive performance of prediction for drug-target interaction, with a concordance of 85.83%, a sensitivity of 79.62% and a specificity of 92.76%.

In this work, the candidate targets were selected according to the criteria that the possibility of interacting with potential candidate targets was higher than 0.6 for the RF model and 0.7 for the SVM model. The obtained candidate targets were finally reserved and were further predicted for their targets.

### 3.4. Target Validation

To validate the drug-target binding, molecular docking analysis was carried out using the AutoDock software (available online: http://autodock.scripps.edu/; accessed on 1 February 2012). This approach performs the docking of the small, flexible ligand to a set of grids describing the target protein [57]. During the docking process, the protein was considered as rigid and the molecules as flexible. All other parameters were adopted as default values. The crystal structures of the candidate targets were downloaded from the RCSB Protein Data Bank (available online: http://www.pdb.org/; accessed on 1 December 2011), and the proteins without crystal structures were performed based on a homology modeling using the Swiss-Model Automated Protein Modelling Server (available online: http://swissmodel.expasy.org/; accessed on 1 February 2012). Target docking scores ≤−5.0 kcal/mol were selected to construct networks.

### 3.5. Network Construction

The target-disease and target-pathway networks were generated by Cytoscape [58], implemented in the TcmSP. In the visualized network, targets that related diseases/signal pathways are represented as nodes and intermolecular interactions. The information about the diseases and pathways were obtained from the TcmSP, which are related with the PharmGkb database (available online: http://www.pharmgkb.org; accessed on 1 December 2011). The “target-disease network” was constructed by linking the protein targets and related diseases, while the “target-pathway network” was built by connecting the targets and their related signal systems. The quantitative properties of these networks were also analyzed by two plugins included in TcmSP.

## 4. Conclusions

TCM is a heritage that is thousands of years old and is still used by millions of people all over the world—even after the development of modern scientific medicine. Chinese herbal combinations generally include one or more plants and even animal products. This creates obstacles in identification of which ingredients in the Chinese herbs are active substances, as well as in the discovery of their targets. Additionally, the mechanism on the molecular/systems level of one of the most important theories in TCM, i.e. “multiple herbal drugs for one disease”, is still not clear.

In this work, we have constructed an integrated model of systems pharmacology by combining the knowledge of chemistry, biology and the theoretical background of TCM to investigate the mechanisms of action of Chinese herbs related to CVD. The obtained results show that the Chinese herbs *Ligusticum chuanxiong*, *Dalbergia odorifera* and *Corydalis yanhusuo* possess 64 bioactive ingredients in total, which significantly inhibit platelet aggregation and inflammation, and also exhibit great spasmolytic effects.

For the first time, we identified 54 protein targets, which are closely associated with CVD for the three Chinese herbs, of which 29 are common targets (52.7%), which clarifies the mechanism of efficiency of the herbs for the treatment of CVD. Also, this result indicates that the use of multiple drugs enables targeting the same targets simultaneously and thus exhibiting similar pharmacological effects on one disease. This helps to elucidate the mechanisms of “multiple herbal drugs for one disease”, one of the most important doctrines in TCM. This will provide more clues for the treatment of complex diseases and design of new combined drugs.

## Supplementary Materials



## Figures and Tables

**Table 1:** All 64 compounds in three herbs and the corresponding predicted oral bioavailability (OB).

No.	Molecule	OB	Herbs *
M8	Carotol	149.03	Lc
M20	1-Acetyl-carboline	67.12	Lc
M30	2,3,5-Trihydroxymethyl-6-methylpyrazine	73.44	Lc
M43	3,4-Dihydroxybenzoic acid	40.10	Lc
M53	4,7-Dihydroxy-3-butylphthalide	105.83	Lc
M54	4-hydroxy-3-butylphthalide	63.44	Lc
M57	5-hydroxymethyl-3,6-dimethylpyrazine-2-methanioc-acid	59.09	Lc
M59	7-oxabicyclo-2/1,4-Cineole	60.87	Lc
M70	Aromadendrene-oxide-2	64.71	Lc
M82	Bornyl-acetate	65.53	Lc
M85	3-*n*-Butylphthalide	71.28	Lc
M92	*Cis*-sabinenehydrate	94.32	Lc
M93	Cnidilide	77.55	Lc
M105	Ferulic acid	86.58	Lc
M106	Folic-acid	70.51	Lc
M112	Globulol-(−)-	81.85	Lc
M120	Ligustilide	50.10	Lc
M121	Ligustrazine	29.64	Lc
M151	Senkyunolide-D	83.37	Lc
M158	Senkyunolide-K	61.86	Lc
M169	Spathulenol	82.37	Lc
M194	Vanillic-acid	64.27	Lc
M285	Vestitone	78.21	Lc
M287	Xenognosin-B	73.99	Lc
M198	(3R)-vestitol	71.33	Do
M217	2′,6-dihydroxy-4′-methoxy-2-arylbenzofuran	67.09	Do
M219	2′-*O*-methylisoliquiritigenin	79.15	Do
M220	3′,4′,7-Trihydroxyflavone	67.84	Do
M241	Butein	78.38	Do
M242	Butin	52.91	Do
M246	Clausseguinone	60.89	Do
M247	Dalbergin	78.57	Do
M250	Duartin	61.17	Do
M256	Hydroxyobtustyrene	78.44	Do
M259	Isoliquiritigenin	61.38	Do
M260	Isomucronustyrene	78.92	Do
M261	Koparin	68.87	Do
M262	Liquiritigenin	67.19	Do
M264	Melilotocarpan-A	87.37	Do
M265	Melilotocarpan-C	78.88	Do
M267	Methyl-2-hydroxy-3,4-dimethoxybenzoate	89.23	Do
M268	Methylnissolin	66.53	Do
M269	Mucronulatol	64.93	Do
M274	Odoricarpin	60.17	Do
M275	Odoriflavene	84.49	Do
M280	Prunetin	43.44	Do
M281	Sativanone	73.01	Do
M286	Violanone	71.83	Do
M297	Bicuculline	76.96	Do
M263	Medicarpin	76.02	Cy
M292	13-methyldehydrocorydalmine	72.22	Cy
M293	13-methylpalmatrubine	70.97	Cy
M300	Capaurine	71.12	Cy
M301	Caseanidine	93.87	Cy
M303	Clarkeanidine	92.55	Cy
M311	Corynoloxine	68.61	Cy
M312	Coryphenanthrine	61.15	Cy
M316	Dehydrocavidine	47.59	Cy
M318	Dehydrocorydaline	60.36	Cy
M338	Norglaucine	61.25	Cy
M346	Pseudoprotopine	63.63	Cy
M356	Tetrahydropalmatine	75.78	Cy
M357	Tetrahydroprotopapaverine	64.29	Cy
M360	Yuanhunine	84.94	Cy

* *Ligusticum chuanxiong* (Lc), *Dalbergia odorifera* (Do) and *Corydalis yanhusuo* (Cy).

## References

[1] Knutsson A., Boggild H. (2000). Shiftwork and cardiovascular disease: Review of disease mechanisms. Rev. Environ. Health.

[2] Xu J., Wu H.Y. (2009). Chinese herbal medicine and acupuncture for the treatment of cardiovascular disease. J. Geriatr. Cardiol.

[3] Cheung F. (2011). TCM: Made in China. Nature.

[4] Tachjian A., Maria V., Jahangir A. (2010). Use of herbal products and potential interactions in patients with cardiovascular diseases. J. Am. Coll. Cardiol.

[5] Ran X., Ma L., Peng C., Zhang H., Qin L.-P. (2011). *Ligusticum chuanxiong* Hort: A review of chemistry and pharmacology. Pharm. Biol.

[6] Liang M.-J., He L.-C., Yang G.-D. (2005). Screening, analysis and *in vitro* vasodilatation of effective components from *Ligusticum chuanxiong*. Life Sci.

[7] Tao Y., Wang Y. (2010). Bioactive sesquiterpenes isolated from the essential oil *of Dalbergia odorifera* T. Chen. Fitoterapia.

[8] Steimle A.E., Lange R.A., Hillis L.D. (2004). Antiplatelet therapy for ischemic heart disease. N. Engl. J. Med.

[9] Liu J.L., Liu H. (1994). The protective effects of dl-tetrahydropalmatine on isolated rat heart against ischemic/reperfusion damage exacerbated by extrinsic radical generating system. Zhongguo Yaoxue Zazhi.

[10] Ling H., Wu L., Li L. (2006). *Corydalis yanhusuo* rhizoma extract reduces infarct size and improves heart function during myocardial ischemia/reperfusion by inhibiting apoptosis in rats. Phytother. Res.

[11] Yuan C.-S., Mehendale S.R., Wang C.-Z., Aung H.H., Jiang T., Guan X., Shoyama Y. (2004). Effects of *Corydalis yanhusuo* and *Angelicae dahuricae* on cold pressor-induced pain in humans: A controlled trial. J. Clin. Pharmacol.

[12] Li X.X., Wang J.N., Yu H., Yang H.J., Xu H.Y., Tang S.H., Li Y., Wang Y.H., Yang L., Huang L.Q. (2012). Investigation into the mechanisms of action of traditional Chinese medicine from chemical, genomic and pharmacological data in an integrated framework. PLoS One.

[13] Yu H., Chen J.X., Xu X., Li Y., Zhao H.H., Fang Y.P., Li X.X., Zhou W., Wang W., Wang Y.H. (2012). A Systematic prediction of multiple drug-target interactions from chemical, genomic and pharmacological data. PLoS One.

[14] Chan L.M.S., Lowes S., Hirst B.H. (2004). The ABCs of drug transport in intestine and liver: Efflux proteins limiting drug absorption and bioavailability. Eur. J. Pharm. Sci.

[15] Yan R., Ko N.L., Li S.L., Tam Y.K., Lin G. (2008). Pharmacokinetics and metabolism of ligustilide, a major bioactive component in *Rhizoma chuanxiong*, in the rat. Drug Metab. Dispos.

[16] Ko W.C. (1980). A newly isolated antispasmodic butylidenephthahde. Jpn. J. Pharmacol.

[17] Wei Y., Hu J., Li H., Liu J.G. (2011). Preparative isolation and purification of senkyunolide-I, senkyunolide-H and ferulic acid from *Rhizoma chuanxiong* using counter-current chromatography. J. Sep. Sci.

[18] Che M.T., Wen Y.C., Wun C.K., Ouyang C.H. (1987). Antiplatelet effect of butylidenephthalide. Biochim. Biophys. Acta Gen. Subj.

[19] Ozaki Y., Sekita S., Harada M. (1989). Centrally acting muscle relaxant effect of phthalides (ligustilide, cnidilide and senkyunolide) obtained from *Cnidium officinale* Makino. Yakugaku Zasshi.

[20] Jasicka-Misiak I., Lipok J., Nowakowska E.M., Wieczorek P.P., Mlynarz P., Kafarski P. (2004). Antifungal activity of the carrot seed oil and its major sesquiterpene compounds. Z. Naturforsch. C J. Biosci.

[21] Graf E. (1992). Antioxidant potential of ferulic acid. Free Radic. Biol. Med.

[22] Hou Y.Z., Yang J., Zhao G.R., Yuan Y.J. (2004). Ferulic acid inhibits vascular smooth muscle cell proliferation induced by angiotensin II. Eur. J. Pharmacol.

[23] Zhao Z.H., Moghadasian M.H. (2008). Chemistry, natural sources, dietary intake and pharmacokinetic properties of ferulic acid: A review. Food Chem.

[24] Jackson S.P. (2007). The growing complexity of platelet aggregation. Blood.

[25] Yadav V.R., Prasad S., Sung B., Aggarwal B.B. (2011). The role of chalcones in suppression of NF-κB-mediated inflammation and cancer. Int. Immunopharmacol.

[26] Kim Y.W., Zhao R.J., Park S.J., Lee J.R., Cho I.J., Yang C.H., Kim S.G., Kim S.C. (2008). Anti-inflammatory effects of liquiritigenin as a consequence of the inhibition of NF-κB-dependent iNOS and proinflammatory cytokines production. Br. J. Pharmacol.

[27] Yu X.L., Wang W., Yang M. (2007). Antioxidant activities of compounds isolated from *Dalbergia odorifera* T. Chen and their inhibition effects on the decrease of glutathione level of rat lens induced by UV irradiation. Food Chem.

[28] Lin W.W., Karin M. (2007). A cytokine-mediated link between innate immunity, inflammation, and cancer. J. Clin. Invest.

[29] Aggarwal B.B., Shishodia S., Sandur S.K., Pandey M.K., Sethi G. (2006). Inflammation and cancer: How hot is the link?. Biochem. Pharmacol.

[30] Ding B., Zhou T., Fan G., Hong Z., Wu Y. (2007). Qualitative and quantitative determination of ten alkaloids in traditional Chinese medicine *Corydalis yanhusuo* W.T. Wang by LC-MS/MS and LC-DAD. J. Pharm. Biomed. Anal.

[31] Khan I.A., Alam S.S., Haq A., Jabbar A. (2005). Biochemistry of resistance in chickpea against wilt disease caused by *Fusarium osxysporum* F.SP. ciceris. Pak. J. Bot.

[32] Maurya S., Srivastava J.S., Jha R.N., Panday V.B., Singh U.P. (2001). Effect of tetrahydropalmatine, an alkaloid on spore germination of some fungi. Mycobiology.

[33] Kurahashi K., Fujiwara M. (1976). Adrenergic neuron blocking action of dehydrocorydaline isolated from *Corydalis bulbosa*. Can. J. Physiol. Pharmacol.

[34] Li H.L., Han T., Liu R.H., Zhang C., Chen H.S., Zhang W.D. (2008). Alkaloids from *Corydalis saxicola* and their anti-hepatitis B virus activity. Chem. Biodivers.

[35] Gray G.A., Webb D.J. (1996). The endothelin system and its potential as a therapeutic target in cardiovascular disease. Pharmacol. Ther.

[36] Douglas S.A., Ohlstein E.H. (2000). Human urotensin-II, the most potent mammalian vasoconstrictor identified to date, as a therapeutic target for the management of cardiovascular disease. Trends Cardiovasc. Med.

[37] Linsel-Nitschke P., Tall A.R. (2005). HDL as a target in the treatment of atherosclerotic cardiovascular disease. Nat. Rev. Drug Discov.

[38] Moens A.L., Kass D.A. (2006). Tetrahydrobiopterin and cardiovascular disease. Arterioscler. Thromb. Vasc. Biol.

[39] Liu P.Y., Death A.K., Handelsman D.J. (2003). Androgens and cardiovascular disease. Endocr. Rev.

[40] Yusuf S., Sleight P., Pogue J., Bosch J., Davies R., Dagenais G. (2000). Effects of an angiotensin-converting-enzyme inhibitor, ramipril, on cardiovascular events in high-risk patients. N. Engl. J. Med.

[41] Sun D.Z., Xu L., Wei P.K., Liu L., He J. (2007). Syndrome differentiation in traditional Chinese medicine and E-cadherin/ICAM-1 gene protein expression in gastric carcinoma. World J. Gastroenterol.

[42] Hishikawa K., Nakaki T., Fujii T. (2000). Connective tissue growth factor induces apoptosis via caspase 3 in cultured human aortic smooth muscle cells. Eur. J. Pharmacol.

[43] Lee K.H., Itokawa H., Kozuka M, Ho C.T., Lin J.K., Zheng Q.Y. (2003). Oriental Herbal Products: The Basis for Development of Dietary Supplements and New Medicines in the 21st Century. Oriental Foods and Herbs.

[44] Small K.M., Wagoner L.E., Levin A.M., Kardia S.L.R., Liggett S.B. (2002). Synergistic polymorphisms of b1-and a 2C-adrenergic receptors and the risk of congestive heart failure. N. Engl. J. Med.

[45] Kelly H.B. (1979). Coronary artery disease in aviation. Routine examination of aircrew. J. R. Soc. Med.

[46] Miles E.A., Zoubouli P., Calder P.C. (2005). Differential anti-inflammatory effects of phenolic compounds from extra virgin olive oil identified in human whole blood cultures. Nutrition.

[47] Cheng W.H., Lu P.J., Ho W.Y., Tung C.S., Cheng P.W., Hsiao M., Tseng C.J. (2010). Angiotensin II inhibits neuronal nitric oxide synthase activation through the ERK1/2-RSK signaling pathway to modulate central control of blood pressure. Circ. Res.

[48] Grosser T., Fries S., FitzGerald G.A. (2006). Biological basis for the cardiovascular consequences of COX-2 inhibition: therapeutic challenges and opportunities. J. Clin. Invest.

[49] Houslay M.D., Baillie G.S., Maurice D.H. (2007). cAMP-Specific phosphodiesterase-4 enzymes in the cardiovascular system: A molecular toolbox for generating compartmentalized cAMP signaling. Circ. Res.

[50] Xu Q. (2002). Role of heat shock proteins in atherosclerosis. Arterioscler. Thromb. Vasc. Biol.

[51] Atlas S.A. (2007). The renin-angiotensin aldosterone system: Pathophysiological role and pharmacologic inhibition. J. Manag. Care Pharm.

[52] Chou M.T., Wang J., Fujita D.J. (2002). c kinase becomes preferentially associated with the VEGFR, KDR/Flk-1, following VEGF stimulation of vascular endothelial cells. BMC Biochem.

[53] Bouloumie A., Schini-Kerth V.B., Busse R. (1999). Vascular endothelial growth factor up-regulates nitric oxide synthase expression in endothelial cells. Cardiovasc. Res.

[54] Fontana J., Fulton D., Chen Y., Fairchild T.A., McCabe T.J., Fujita N., Tsuruo T., Sessa W.C. (2002). Domain mapping studies reveal that the M domain of hsp90 serves as a molecular scaffold to regulate Akt-dependent phosphorylation of endothelial nitric oxide synthase and NO release. Circ. Res.

[55] Traditional Chinese Medicine Systems Pharmacology Database and Analysis Platform.

[56] Xu X., Zhang W.X., Li Y., Huang C., Yu H., Wang Y.H., Duan J.Y. (2012). *In silico* prediction of human oral bioavailability based on molecular properties integrated with metabolism. Int. J. Mol. Sci.

[57] Cosconati S., Forli S., Perryman A.L., Harris R., Goodsell D.S., Olson A.J. (2010). Virtual screening with autodock: Theory and practice. Expert Opin. Drug Discov.

[58] Smoot M.E., Ono K., Ruscheinski J., Wang P.L., Ideker T. (2011). Cytoscape 2.8: New features for data integration and network visualization. Bioinformatics.

