# Supplementary material for: A Systems Biology Approach to Understanding the Mechanisms of Action of Chinese Herbs for Treatment of Cardiovascular Disease
Source: Int J Mol Sci. 2012 Oct 19;13(10):13501–20. doi: 10.3390/ijms131013501 (PMC3497338; doi:10.3390/ijms131013501)
Supplement: Supplementary file 1 [file ijms-13-13501-s001.pdf]

Supplementary Information

Table S1. All 360 compounds in the three herbs and the corresponding predicted oral bioavailability (OB), and CAS number.

No.	Molecule	CAS	OB (%)	Herbs *
M1	(-)-alpha-cubebene	17699-14-8	32.62	Lc
M2	(-)-beta-Elemene	515-13-9	25.97	Lc
M3	(-)-isoledene	95910-36-4	53.02	Lc
M4	(-)-p-mentha-1,5-diene	4221-98-1	26.62	Lc
M5	(-)-spathulenol	77171-55-2	27.88	Lc
M6	(+)-alpha-funebrene	50894-66-1	52.87	Lc
M7	(1alpha,2beta,4beta)-1-methyl-2,4-bis(methylvinyl)-1-vinylcyclohexane	33880-83-0	24.44	Lc
M8	carotol	465-28-1	149.03	Lc
M9	(E,E)-undeca-1,3,5-triene	19883-29-5	18.18	Lc
M10	(Z）-6,8’,7,3’-diligustilide	-	11.96	Lc
M11	(Z,Z)-9,12-Octadecadien-1-ol	506-43-4	37.76	Lc
M12	1-(2-Hydroxy-5-methylphenyl)ethanone	1450-72-2	26.65	Lc
M13	1,1-Diethoxybutane	3658-95-5	29.65	Lc
M14	1,1-dimethylcyclopentane	1638-26-2	41.22	Lc
M15	1,2,3,4,4a,7-Hexahydro-l,6-dimethyl-4-(1-methylethyl)-naphthalene	-	19.06	Lc
M16	1,3,6-heptatriene,2,5,5-trimethyl-	29548-02-5	42.10	Lc
M17	1,3,8-p-Menthatriene	18368-95-1	35.91	Lc
M18	1,5,5-Trimethyl-6-methylenecyclohexene	514-95-4	46.08	Lc
M19	1-acetyl-2-phenylhydrazine	114-83-0	21.20	Lc
M20	1-Acetyl-carboline	50892-83-6	67.12	Lc
M21	1-beta-ethylacrylate-7-aldehyde-beta-carboline	-	28.77	Lc
M22	1-Nonanal	124-19-6	40.19	Lc
M23	1-Octanol,2,7-dimethyl-	-	22.95	Lc
M24	1-propanone,1-[2-furanyl]-	3194-15-8	63.69	Lc
M25	1-propanone,1-phenyl-	93-55-0	60.17	Lc
M26	1-Terpineol	586-82-3	49.77	Lc
M27	2-(1-oxopentyl)-benzoic acid methyl ester	-	58.32	Lc
M28	2,2,3-trimethyl-3-cyclopentene-1-acetaldehyde	4501-58-0	45.18	Lc
M29	2,2,3-Trimethylcyclopent-3-ene-1-carboxaldehyde	33843-21-9	42.64	Lc
M30	2,3,5-trihydroxymethyl-6-methylpyrazine	-	73.44	Lc
M31	2-alyl-3,5,6-trimethylpyrazine	-	29.82	Lc
M32	2-furancarboxylic acid,methyl ester	611-13-2	49.41	Lc
M33	2*H*-pyran-2-one,tetrahydro-5,6-dimethyl-,trans-	24405-16-1	48.04	Lc
M34	2-hydroxymethyl-3,5,6-trimethyl-pyrazine	-	29.66	Lc
M35	2-Methoxy-4-vinylphenol	7786-61-0	41.30	Lc
M36	2-methyl benzoxazole	95-21-6	65.68	Lc
M37	2-methyl-1-propenyl-benzene	768-49-0	34.96	Lc
M38	2-methyl-5-(1-methylene)-1,3-cyclohexadiene	-	40.99	Lc
M39	2-methyl-5-(1-methylethenyl)-2-cyclohexen-1-ol	99-48-9	48.12	Lc
M40	2-methylhexane	40626-78-6	59.94	Lc
M41	2-Pentyl-fura	3777-69-3	54.53	Lc
M42	3(*S*)-3-Butyl-4,5-dihydrophthalide	-	25.99	Lc

M43	3,4-Dihydroxybenzoic acid	99-50-3	70.59	Lc
M44	3,4-epoxy-2,2,7,7-tetramethyl-octane	-	24.21	Lc
M45	3,5,6-trihydroxymethylpyrazine-2-methanoic-acid	-	16.51	Lc
M46	3,5,6-trimethylpyrazine-2-methanoic-acid	-	37.96	Lc
M47	3-carene	13466-78-9	45.15	Lc
M48	3-cyclohexen-1-ol	822-66-2	70.52	Lc
M49	3-furancarboxylic acid,methyl ester	13129-23-2	77.82	Lc
M50	3-methyl-6-(1-methylethyl)-2-cyclohexen-1-ol	491-04-3	47.88	Lc
M51	3-methylene-6-(1-methylethyl)-cyclohexene	555-10-2	40.25	Lc
M52	4-(1-Methylethyl)-1-cyclohexene-1-carboxaldehyde	21391-98-0	40.36	Lc
M53	4,7－Dihydroxy－3－butylphthalide	-	105.83	Lc
M54	4-hydroxy-3-butylphthalide	-	63.44	Lc
M55	4-Iodoindoline	939759-03-2	27.36	Lc
M56	4-octanone	589-63-9	19.37	Lc
M57	5-hydroxymethyl-3,6-dimethylpyrazine-2-methanioc-acid	-	59.09	Lc
M58	6-butyl-1,4-cycloheptadiene	33156-91-1	31.51	Lc
M59	1,4-Cineole	470-67-7	60.87	Lc
M60	Adenine	73-24-5	63.12	Lc
M61	Adenosine	58-61-7	10.03	Lc
M62	alpha-Bergamotene	17699-05-7	15.69	Lc
M63	alpha-Curcumene	644-30-4	23.87	Lc
M64	alpha-phellandrene	99-83-2	27.95	Lc
M65	alpha-pinene	80-56-8	46.25	Lc
M66	alpha-Selinene	473-13-2	31.85	Lc
M67	alpha-Terpinene	99-86-5	32.89	Lc
M68	Alpha-thujene	2867_05_2	48.66	Lc
M69	Amylbenzene	538-68-1	34.30	Lc
M70	aromadendrene oxide 2	85710-39-0	64.71	Lc
M71	aromadendrene,(+)	489-39-4	55.90	Lc
M72	augustic acid	26707-60-8	16.36	Lc
M73	benzene,1-ethyl-3-methyl-	620-14-4	50.77	Lc
M74	benzyl alcohol	100-51-6	60.09	Lc
M75	beta-Acoradiene	28477-64-7	40.01	Lc
M76	beta-cubebene	13744-15-5	15.59	Lc
M77	beta-eudesmene	17066-67-0	23.65	Lc
M78	beta-Pinene	127-91-3	44.84	Lc
M79	beta-Scliene	-	24.21	Lc
M80	beta-Sesquiphellandrene	20307-83-9	17.47	Lc
M81	bicylogermacrene	24703-35-3	22.01	Lc
M82	Bornyl acetate	76-49-3	65.53	Lc
M83	Butanedioic acid, hydroxy-, dimethyl ester, (2R)-	70681-41-3	34.15	Lc
M84	butylidene phthalide	551-08-6	38.84	Lc
M85	Butylphthalide	6066-49-5	71.28	Lc
M86	Caffeic acid	331-39-5	26.00	Lc
M87	Camphene	79-92-5	35.05	Lc
M88	Cedrene	469-61-4	51.14	Lc
M89	chuanxingol	87421-30-5	36.49	Lc
M90	cis-Asarone	5273-86-9	47.30	Lc

M91	cis-Piperitol	16721-38-3	43.19	Lc
M92	cis-sabinenehydrate	546-79-2	94.32	Lc
M93	cnidilide	3674-03-1	77.55	Lc
M94	Coniferylfcrulate	63644-62-2	4.54	Lc
M95	cyclodecene	3618-12-0	47.89	Lc
M96	cyclohexane,1,1,2,3-tetramethyl-	6783-92-2	48.08	Lc
M97	D(+)-Sucrose	57-50-1	3.10	Lc
M98	Decahydro-1，6-bis(methylene)-4-(1-methylethyl)-naphthalene	54932-90-0	28.34	Lc
M99	decane,2,9-dimethyl-	1002-17-1	16.76	Lc
M100	dihydroeugenol	2785-87-7	60.57	Lc
M101	D-Limonene	138-86-3	37.40	Lc
M102	elemol	639-99-6	30.49	Lc
M103	Ethyl linoleate	544-35-4	42.00	Lc
M104	eudesma-4,11-diene	17627-30-4	22.18	Lc
M105	ferulic acid	1135-24-6	86.58	Lc
M106	folic acid	59-30-3	70.51	Lc
M107	frufural	1998-01-1	35.26	Lc
M108	gamma-Bisabolene	495-62-5	18.50	Lc
M109	gamma-Terpinene	99-85-4	34.37	Lc
M110	Germacrene	-	16.42	Lc
M111	Germacrene D	37839-63-7	18.90	Lc
M112	globulol,(-)-	489-41-8	81.85	Lc
M113	Hendecanoic acid	112-37-8	30.25	Lc
M114	heptanal	111-71-7	19.55	Lc
M115	Heptane	142-82-5	41.80	Lc
M116	hexanophenone	942-92-7	51.63	Lc
M117	hinesol	23811-08-7	37.90	Lc
M118	isopropyl phenyl ketone	611-70-1	80.39	Lc
M119	Levistolide-A	88182-33-6	9.48	Lc
M120	Ligustilide	4431-01-0	50.10	Lc
M121	Ligustrazine	1124-11-4	29.64	Lc
M122	linalool	78-70-6	39.51	Lc
M123	L-valyl-L-valinc achydride	350702-73-7	25.61	Lc
M124	Methyl eugenol	93-15-2	37.13	Lc
M125	Methyl linoleate	68605-14-1	41.93	Lc
M126	Methyl oleate	112-62-9	31.90	Lc
M127	Methyl palmitate	112-39-0	18.09	Lc
M128	methyl pentadecanoate	7132-64-1	11.22	Lc
M129	Myrcene	123-35-3	19.01	Lc
M130	myricanone	32492-74-3	59.78	Lc
M131	*n*-Caprylaldehyde	124-13-0	19.07	Lc
M132	neocnidilide	4567-33-3	28.88	Lc
M133	*n*-Hexanal	66-25-1	55.71	Lc
M134	*n*-octatriacontane	7194-85-6	7.91	Lc
M135	octane	111-65-9	29.72	Lc
M136	*o*-Cymene	527-84-4	51.89	Lc
M137	Palmitic acid	1957-10-3	19.30	Lc
M138	*p*-cymene	99-87-6	27.15	Lc

M139	*p*-cymenol	1197-01-9	32.22	Lc
M140	pentadecane	629-62-9	13.97	Lc
M141	Pentyl benzenemethanol	-	55.76	Lc
M142	perlolyrine	29700-20-7	40.02	Lc
M143	*p*-menth-1-en-4-ol	20126-76-5	81.22	Lc
M144	*p*-mentha-1,4(8)-diene	586-62-9	29.72	Lc
M145	pregnenolone	145-13-1	35.67	Lc
M146	Sabinene	3387-41-5	46.14	Lc
M147	sedanoic acid	6697-07-0	44.45	Lc
M148	sedanolide	6415-59-4	21.94	Lc
M149	Senkyunolide A	63038-10-8	26.69	Lc
M150	senkyunolide C	91652-78-7	39.45	Lc
M151	senkyunolide D	94530-82-2	83.37	Lc
M152	senkyunolide E	94530-83-3	18.53	Lc
M153	senkyunolide F	94530-84-4	40.39	Lc
M154	Senkyunolide G	94530-85-5	39.31	Lc
M155	Senkyunolide H	94596-27-7	20.64	Lc
M156	Senkyunolide I	94596-28-8	52.99	Lc
M157	senkyunolide J	94530-86-6	42.15	Lc
M158	Senkyunolide K	114569-33-4	61.86	Lc
M159	senkyunolide L	114586-51-5	30.19	Lc
M160	Senkyunolide M	114569-34-5	3.17	Lc
M161	Senkyunolide N	140694-58-2	36.62	Lc
M162	Senkyunolide O	142797-35-1	10.14	Lc
M163	Senkyunolide P	142864-23-1	9.22	Lc
M164	Senkyunolide Q	142235-81-2	19.41	Lc
M165	Senkyunolide R	172549-37-0	21.38	Lc
M166	senkyunolide S	-	17.01	Lc
M167	senkyunone	142182-61-4	47.66	Lc
M168	sinapic acid	530-59-6	10.67	Lc
M169	spathulenol	6750-60-3	82.37	Lc
M170	Stearic acid	1957-11-4	17.83	Lc
M171	Terpinen-4-ol	562-74-3	81.22	Lc
M172	terpineol	8000-41-7	46.62	Lc
M173	tetradecane	629-59-4	15.93	Lc
M174	thymol	89-83-8	44.03	Lc
M175	trans-2-Nonen-1-ol	31502-14-4	19.93	Lc
M176	trans-ocimene	6874-10-8	22.24	Lc
M177	trans-Piperitol	25437-28-9	47.52	Lc
M178	trimethylamine	75-50-3	59.98	Lc
M179	uracil	66-22-8	42.53	Lc
M180	Valerophenone	1009-14-9	42.58	Lc
M181	Vanillin	121-33-5	52.85	Lc
M182	Verbenene	4080-46-0	37.71	Lc
M183	wallichilide	93236-64-7	5.05	Lc
M184	xiongterpene	-	8.58	Lc
M185	Z-butylidenephthalide	551-08-6	41.80	Lc
M186	Z-Neo-allo-ocimene	673-84-7	14.89	Lc

M187	caryophyllene	6753-98-6	24.77	Lc
M188	farnesene	502-61-4	7.94	Lc
M189	palmitic acid ethyl ester	628-97-7	18.99	Lc
M190	beta-Sitosterol	83-46-5	36.91	Lc
M191	daucosterol	474-58-8	20.63	Lc
M192	linoleic acid	60-33-3	41.90	Lc
M193	oleic acid	112-80-1	33.13	Lc
M194	Vanillic acid	121-34-6	64.27	Lc
M195	(*3R*)-4'-methoxy-2',3,7-trihydroxyisoflavanone	-	43.11	Do
M196	(3*R*)-5'-methoxyvestitol	-	4.20	Do
M197	(3*R*)-7,2',3'-trihydroxy-4-methoxyisoflavan	-	57.20	Do
M198	(3*R*)-vestitol	35878-41-2	71.33	Do
M199	(3*R*,4*R*)-2',3',7-trihydroxy-4'-methoxy-4-[(3*R*)-2',7-dihydroxy-4'-methoxyisoflavan-5'-yl]-isoflavan	-	2.41	Do
M200	(3*R*,4*R*)-2',7-dihydroxy-4',5'-dimethoxy-4-[(3*R*)-2',7-dihydroxy-4'-methoxyisoflavan-5'-yl]isoflavan	-	24.37	Do
M201	(3*R*,4*R*)-3',7-dihydroxy-2',4'-dimethoxy-4-[(2*S*)-4',5,7-trihydroxyflavanone-6-yl]isoflavan	-	39.93	Do
M202	(3*R*,4*R*)-3',7-dihydroxy-2',4'-dimethoxy-4-[(3*R*)-2',7-dihydroxy-4'-methoxyisoflavan-5'-yl]isoflavan	-	1.29	Do
M203	(*R*)-3-(2,3-dihydroxy-4-methoxyphenyl)-7-hydroxychroman-4-one	122587-88-6	48.27	Do
M204	(*R*)-3-(2,3-dihydroxy-4-methoxyphenyl)chroman-7,8-diol	122587-87-5	37.53	Do
M205	(*E*)-3,8-nonadien-2-one	55282-90-1	19.74	Do
M206	(*E*)-beta-farnesene	18794-84-8	7.57	Do
M207	1,2,4-trimethylcyclohexane	2234-75-5	44.68	Do
M208	1-Butanol	71-36-3	91.11	Do
M209	2,2,6-trimethyl-6-vinyltetrahydro-2h-pyran-3-ol	14049-11-7	43.09	Do
M210	2,3-didehydro-2',7-dihydroxy-4'-methoxy-3(2',7-dihydroxy-4'-methoxyisoflavan-5'-yl)flavan	-	32.35	Do
M211	2,3-didehydro-2',7-dihydroxy-4'-methoxy-3-(2',7-dihydroxy-4'-methoxyisoflavan-5'-yl)flavone	-	1.25	Do
M212	2,3-didehydro-2',7-dihydroxy-4'-methoxy-3(2',7-dihydroxy-4'-methoxyisoflavan-6-yl)flavan	-	1.22	Do
M213	2,4-dihydroxy-5-methoxybenzophenone	-	44.19	Do
M214	2,4-dihydroxy-5-methoxyphenylmethanone	-	39.54	Do
M215	2,4-dimethyl-2,4-heptadienal	42452-48-2	57.48	Do
M216	2,4-dimethyl-2,6-heptadienal	80192-48-9	58.28	Do
M217	2',6-dihydroxy-4'-methoxy-2-arylbenzofuran	-	67.09	Do
M218	2-Methyl-6-hydroxyquinoline	613-21-8	45.02	Do
M219	2'-*O*-methylisoliquiritigenin	51828-10-5	79.15	Do
M220	3',4',7-Trihydroxyflavone	2150-11-0	67.84	Do
M221	3-hydroxy-9-methoxy-coumestan	1690-62-6	37.39	Do
M222	3'-hydroxymelanettin	200391-95-3	31.26	Do
M223	3'-methoxydaidzein	21913-98-4	5.37	Do
M224	3-methoxypyridazine	19064-65-4	23.87	Do
M225	3'-*O*-methylviolanone	56973-42-3	49.75	Do
M226	4',5',7-trimethyl-3-methoxyflavone	-	25.94	Do
M227	4-methoxydalbergione	28396-75-0	38.14	Do

M228	4-Methyl-4-hydroxycyclohexanone	17429-02-6	70.25	Do
M229	6,10,14-trimethyl-2-pentadecanone	502-69-2	7.35	Do
M230	7-hydroxy-4'-methoxy-2',5'-dioxo-4-	-	0.82	Do
M231	9-Oxonerolidol	58865-88-6	61.78	Do
M232	alpha-bisabolol	515-69-5	34.65	Do
M233	alpha-santalol	115-71-9	32.79	Do
M234	Alpinetin	1090-65-9	49.63	Do
M235	Benzaldehyde	100-52-7	32.63	Do
M236	benzofuran	271-89-6	34.85	Do
M237	benzothiazole	95-16-9	70.84	Do
M238	beta-angelialacton/5-Methyl-2(5*H*)-furanone	591-11-7	64.19	Do
M239	beta-bisabolene	495-61-4	29.47	Do
M240	bowdichione	53774-75-7	43.19	Do
M241	butein	21849-70-7	78.38	Do
M242	butin	492-14-8	52.91	Do
M243	caryophyllene oxide	1139-30-6	31.64	Do
M244	cearoin	52811-37-7	47.34	Do
M245	chalcone	94-41-7	51.22	Do
M246	clausseguinone	35878-39-8	60.89	Do
M247	dalbergin	482-83-7	78.57	Do
M248	dihydrolinalool	18479-49-7	25.42	Do
M249	D-nerolidol	142-50-7	38.91	Do
M250	duartin	52305-04-1	61.17	Do
M251	Eriodictyol	552-58-9	54.77	Do
M252	eucalyptol	470-82-6	39.73	Do
M253	formononetin	485-72-3	23.44	Do
M254	geranylaceton	3796-70-1	18.69	Do
M255	Guaiacol	90-05-1	48.79	Do
M256	hydroxyobtustyrene	101153-43-9	78.44	Do
M257	indole	120-72-9	34.38	Do
M258	isoduartin	101153-40-6	3.70	Do
M259	isoliquiritigenin	961-29-5	61.38	Do
M260	isomucronustyrene	69471-12-1	78.92	Do
M261	Koparin	65048-75-1	68.87	Do
M262	liquiritigenin	578-86-9	67.19	Do
M263	medicarpin	32383-76-9	76.02	Do
M264	Melilotocarpan A	61135-95-3	87.37	Do
M265	melilotocarpan C	83013-82-5	78.88	Do
M266	melilotocarpan D	83013-81-4	39.35	Do
M267	methyl 2-hydroxy-3,4-dimethoxybenzoate	6395-23-9	89.23	Do
M268	Methylnissolin	73340-41-7	66.53	Do
M269	mucronulatol	20878-98-2	64.93	Do
M270	Myristic acid	544-63-8	14.24	Do
M271	naringenin	480-41-1	41.08	Do
M272	*n*-hexadecanoic acid	57-10-3	19.30	Do
M273	obtustyrene	21148-31-2	8.46	Do
M274	odoricarpin	101153-42-8	60.17	Do
M275	odoriflavene/7,2'-Dihydroxy-3',4'-dimethoxyisoflav-3-ene	101153-41-7	84.49	Do

M276	Phenol	108-95-2	36.05	Do
M277	phytol	102608-53-7	33.82	Do
M278	pinene	80-56-8	46.20	Do
M279	pinocembrin	68745-38-0	45.43	Do
M280	prunetin	552-59-0	43.44	Do
M281	sativanone	70561-31-8	73.01	Do
M282	Stevein	36286-69-8	40.29	Do
M283	Styrene	100-42-5	29.55	Do
M284	Turmerol	82508-15-4	48.49	Do
M285	Vestitone	66211-83-4	78.21	Do
M286	vistitone	52250-38-1	71.83	Do
M287	Xenognosin B	1890-99-9	73.99	Do
M288	(-)-alpha-*N*-methylcanadine	47474-49-7	50.94	Cy
M289	(-)-scoulerine	6451-73-6	37.28	Cy
M290	(*S*)-Isocorypalmine	483-34-1	35.80	Cy
M291	10-nonacosanol	2606-50-0	11.33	Cy
M292	13-methyldehydrocorydalmine	-	72.22	Cy
M293	13-methylpalmatrubine	-	70.97	Cy
M294	Acetylcorynoline	18797-80-3	28.54	Cy
M295	alpha-allocryptopine	24240-04-8	47.39	Cy
M296	berberine	2086-83-1	39.61	Cy
M297	bicuculline	485-49-4	76.96	Cy
M298	Bulbocapnine	298-45-3	54.81	Cy
M299	Canadine	522-97-4	47.27	Cy
M300	Capaurine	478-14-8	71.12	Cy
M301	caseanidine	40141-47-7	93.87	Cy
M302	cavidine	32728-75-9	26.21	Cy
M303	clarkeanidine	99615-99-3	92.55	Cy
M304	columbamine	3621-36-1	35.41	Cy
M305	coptisine	3486-66-6	51.92	Cy
M306	corybulbine	518-77-4	30.11	Cy
M307	corydaline	518-69-4	26.35	Cy
M308	Corydalmine	30413-84-4	7.89	Cy
M309	Corydine	476-69-7	26.81	Cy
M310	corynoline	18797-79-0	31.25	Cy
M311	Corynoloxine	31470-65-2	68.61	Cy
M312	coryphenanthrine	66190-61-2	61.15	Cy
M313	Corytuberine	517-56-6	30.46	Cy
M314	cryptopine	482-74-6	26.77	Cy
M315	d-(+)-tetrahydropalmatine	3520-14-7	49.80	Cy
M316	dehydrocavidine	83218-34-2	47.59	Cy
M317	Dehydrocorybulbine	-	10.22	Cy
M318	dehydrocorydaline	30045-16-0	60.36	Cy
M319	Dehydrocorydalmine	6877-27-6	10.17	Cy
M320	dehydroglaucine	-	29.09	Cy
M321	dehydronantenine	98-15-2	41.01	Cy
M322	demethylcorydalmatine	-	39.23	Cy
M323	Dihydrochelerythrine	6880-91-7	30.80	Cy

M324	Dihydrosanguinarine	3606-45-9	46.95	Cy
M325	Docosanoic acid	112-85-6	15.69	Cy
M326	Emodin	518-82-1	24.54	Cy
M327	Emodin-3-methyl ether	521-61-9	18.59	Cy
M328	Fumaricine	24181-77-9	43.85	Cy
M329	glaucine	475-81-0	23.27	Cy
M330	hydroxystreptomycin	6835-00-3	0.41	Cy
M331	isoboldine	3019-51-0	31.06	Cy
M332	isocorybulbine	22672-74-8	42.04	Cy
M333	leonticine	2609-29-2	49.00	Cy
M334	lirioferine	6883-42-7	14.98	Cy
M335	Mecambridine	31098-60-9	24.84	Cy
M336	nantenine	2565-01-7	14.29	Cy
M337	N-methyllaurotetanine	2169-44-0	28.47	Cy
M338	norglaucine	21848-62-4	61.25	Cy
M339	noroxyhydrastinine	21796-14-5	39.93	Cy
M340	oxoglaucine	5574-24-3	23.76	Cy
M341	palmatine	3486-67-7	29.55	Cy
M342	p-hydroxybenzoic acid	99-96-7	43.75	Cy
M343	pontevedrine	34647-65-9	26.97	Cy
M344	protopine	130-86-9	5.20	Cy
M345	pseudocoptisine	19716-67-7	41.53	Cy
M346	pseudoprotopine	24240-05-9	63.63	Cy
M347	quercetin	117-39-5	45.91	Cy
M348	sanguidimerine	41758-45-6	28.76	Cy
M349	sanguinarine	2447-54-3	43.04	Cy
M350	saulatine	91897-61-9	51.80	Cy
M351	spallidamine	129388-65-4	4.84	Cy
M352	stigmasterol	83-48-7	9.73	Cy
M353	stylopine	7461-02-1	54.12	Cy
M354	tetrahydrocoptisine	4312-32-7	54.12	Cy
M355	Tetrahydrocorysamine	32043-26-8	38.40	Cy
M356	tetrahydropalmatine	10097-84-4	75.78	Cy
M357	tetrahydroprotopapaverine	26193-25-9	64.29	Cy
M358	thalicmidine	5083-88-5	26.44	Cy
M359	Yuanamide	102421-42-1	44.66	Cy
M360	yuanhunine	104387-15-7	84.94	Cy

* *Ligusticum chuanxiong* (Lc), *Dalbergia odorifera*(Do) and *Corydalis yanhusuo*(Cy)

Table S2. The information of all 261 protein targets.

No.	Protein name
P4 *	Voltage-dependent L-type calcium channel subunit alpha-1C
P8 *	Tyrosine-protein phosphatase non-receptor type 1
P9 *	Tyrosine-protein kinase JAK2
P10 *	Tumor necrosis factor
P25 *	Sodium channel protein type 4 subunit alpha
P28 *	Serine/threonine-protein kinase Chk1
P34 *	Retinoic acid receptor gamma-1
P35 *	Renin
P41 *	Proto-oncogene tyrosine-protein kinase Src
P45 *	Prothrombin
P46 *	Prostaglandin G/H synthase 2
P47 *	Prostaglandin G/H synthase 1
P51 *	Potassium voltage-gated channel subfamily H member 2
P53 *	Phospholipase A2, membrane associated
P55 *	Phosphatidylinositol-4,5-bisphosphate 3-kinase catalytic subunit gamma isoform
P57 *	Peroxisome proliferator-activated receptor gamma
P58 *	Peroxisome proliferator-activated receptor delta
P59 *	Peroxisome proliferator-activated receptor alpha
P63 *	Orphan nuclear receptor PXR
P66 *	Nitric-oxide synthase, endothelial
P68 *	Nitric oxide synthase, inducible
P72 *	Neuronal acetylcholine receptor subunit alpha-7
P75 *	Neuronal acetylcholine receptor subunit alpha-3
P79 *	NAD(P)H dehydrogenase [quinone] 1
P98 *	Leukotriene A-4 hydrolase
P108 *	Heat shock protein HSP 90-alpha
P110 *	Glycogen synthase kinase-3 beta
P111 *	Glycogen phosphorylase, muscle form
P116 *	Glucocorticoid receptor
P134 *	FK506-binding protein 1A
P136 *	Estrogen receptor beta
P137 *	Estrogen receptor
P150 *	Delta-type opioid receptor
P167 *	Coagulation factor X
P168 *	Coagulation factor VII
P175 *	cGMP-specific 3',5'-cyclic phosphodiesterase
P176 *	cGMP-inhibited 3',5'-cyclic phosphodiesterase A
P183 *	Catechol O-methyltransferase
P184 *	Caspase-3
P186 *	Carboxypeptidase B
P187 *	Carbonic anhydrase 7
P188 *	Carbonic anhydrase 4
P193 *	cAMP-specific 3',5'-cyclic phosphodiesterase 4A
P203 *	Beta-1 adrenergic receptor
P208 *	Arachidonate 5-lipoxygenase

P209 *	Angiotensin-converting enzyme
P210 *	Androgen receptor
P216 *	Alpha-2A adrenergic receptor
P217 *	Alpha-1D adrenergic receptor
P226 *	Adenosine A1 receptor
P252 *	Coagulation factor XI
P260 *	ATP-sensitive inward rectifier potassium channel 11
P261 *	Translocator protein
P262 *	Hemoglobin subunit beta
P1	Voltage-dependent T-type calcium channel subunit alpha-1I
P2	Voltage-dependent T-type calcium channel subunit alpha-1G
P3	Voltage-dependent L-type calcium channel subunit alpha-1S
P5	Voltage-dependent calcium channel subunit alpha-2/delta-1
P6	Vitamin K epoxide reductase complex subunit 1
P7	Urokinase-type plasminogen activator
P11	Trypsin-3
P12	Trypsin-1
P13	Trace amine-associated receptor 1
P14	Thyroid hormone receptor beta-1
P15	Thymidylate synthase
P16	Thymidine kinase
P17	Thermolysin
P18	Synaptic vesicular amine transporter
P19	Stromelysin-1
P20	Streptavidin
P21	Sodium-dependent serotonin transporter
P22	Sodium-dependent noradrenaline transporter
P23	Sodium-dependent dopamine transporter
P24	Sodium channel protein type 5 subunit alpha
P26	Sodium channel protein type 10 subunit alpha
P27	Sigma 1-type opioid receptor
P29	Serine/threonine-protein kinase 6
P30	Serine protease hepsin
P31	Salivary alpha-amylase
P32	Ribosyldihydronicotinamide dehydrogenase [quinone]
P33	Retinoic acid receptor RXR-beta
P36	Reaction center protein M chain
P37	Reaction center protein L chain
P38	Queuine tRNA-ribosyltransferase
P40	Purine nucleoside phosphorylase
P42	Proto-oncogene tyrosine-protein kinase LCK
P43	Proto-oncogene tyrosine-protein kinase ABL1
P44	Proto-oncogene serine/threonine-protein kinase Pim-1
P48	Progesterone receptor
P49	Potassium voltage-gated channel subfamily H member 7
P50	Potassium voltage-gated channel subfamily H member 6
P52	POSSIBLE CELLULASE CELA1
P54	Phospholipase A2

P56	Phenylethanolamine N-methyltransferase
P60	Pepsin A
P61	Pancreatic alpha-amylase
P62	P2Y purinoceptor 12
P64	Nuclear receptor coactivator 2
P65	Nuclear receptor coactivator 1
P67	Nitric-oxide synthase, brain
P69	Nicotinate-nucleotide--dimethylbenzimidazole phosphoribosyltransferase
P70	Neutrophil gelatinase-associated lipocalin
P71	Neutrophil collagenase
P73	Neuronal acetylcholine receptor subunit alpha-6
P74	Neuronal acetylcholine receptor subunit alpha-4
P76	Neuronal acetylcholine receptor subunit alpha-2
P77	Neuronal acetylcholine receptor subunit alpha-10
P78	Neuraminidase
P80	Mu-type opioid receptor
P81	Muscarinic acetylcholine receptor M5
P82	Muscarinic acetylcholine receptor M4
P83	Muscarinic acetylcholine receptor M3
P84	Muscarinic acetylcholine receptor M2
P85	Muscarinic acetylcholine receptor M1
P86	Mitogen-activated protein kinase 8
P87	Mitogen-activated protein kinase 14
P88	Mitogen-activated protein kinase 10
P89	Mitogen-activated protein kinase 1
P90	Methionine aminopeptidase 2
P91	Methionine aminopeptidase
P92	Matrix protein 2
P93	Matrix metalloproteinase-9
P94	MAP kinase-activated protein kinase 2
P95	Macrophage migration inhibitory factor
P96	Macrophage metalloelastase
P97	Lysozyme
P99	Lactotransferrin
P100	Kinesin-like protein KIF11
P101	Kappa-type opioid receptor
P102	Ig kappa chain C region
P103	Ig gamma-2 chain C region
P104	Ig gamma-1 chain C region
P105	Hypoxanthine-guanine phosphoribosyltransferase
P106	Histamine H1 receptor
P107	Heat shock protein HSP 90-beta
P109	Group IIE secretory phospholipase A2
P112	Glutathione-requiring prostaglandin D synthase
P113	Glutamate receptor, ionotropic kainate 2
P114	Glutamate receptor 2
P115	Glutamate [NMDA] receptor subunit 3A
P117	Glucan 1,4-alpha-maltohexaosidase

P118	Glucan 1,3-beta-glucosidase
P119	Genome polyprotein
P120	Gamma-aminobutyric-acid receptor subunit beta-3
P121	Gamma-aminobutyric-acid receptor subunit beta-2
P122	Gamma-aminobutyric-acid receptor subunit alpha-6
P123	Gamma-aminobutyric-acid receptor subunit alpha-5
P124	Gamma-aminobutyric-acid receptor subunit alpha-4
P125	Gamma-aminobutyric-acid receptor subunit alpha-3
P126	Gamma-aminobutyric-acid receptor subunit alpha-2
P127	Gamma-aminobutyric-acid receptor subunit alpha-1
P128	Gamma-aminobutyric acid receptor subunit gamma-3
P129	Gamma-aminobutyric acid receptor subunit gamma-2
P130	Gamma-aminobutyric acid receptor subunit gamma-1
P131	Gamma-aminobutyric acid receptor subunit epsilon
P132	Gag-Pol polyprotein
P133	Fructose-1,6-bisphosphatase 1
P135	Estrogen-related receptor gamma
P138	Ephrin type-B receptor 4
P139	Eosinophil lysophospholipase
P140	Enoyl-[acyl-carrier-protein] reductase [NADH]
P141	Endothelin-1 receptor
P142	Endoglucanase G
P143	Endoglucanase 5A
P144	DNA topoisomerase 2-alpha
P145	DNA topoisomerase 1
P146	Dipeptidyl peptidase 4
P147	Dihydroorotate dehydrogenase, mitochondrial
P148	Dihydrofolate reductase-like protein 1
P149	Dihydrofolate reductase
P151	D-amino-acid oxidase
P152	D(4) dopamine receptor
P153	D(3) dopamine receptor
P154	D(2) dopamine receptor
P155	D(1B) dopamine receptor
P156	D(1A) dopamine receptor
P157	Cytochrome P450-cam
P158	Cytochrome c1, heme protein, mitochondrial
P159	Cytochrome b-c1 complex subunit 8
P160	Cytochrome b-c1 complex subunit 6, mitochondrial
P161	Cytochrome b
P162	Cysteinyl leukotriene receptor 1
P163	Cyclomaltodextrin glucanotransferase
P164	Cyclin-A2
P165	Corticosteroid 11-beta-dehydrogenase isozyme 1
P166	Collagenase 3
P169	Chymotrypsinogen B
P170	Chymotrypsin-like elastase family member 1
P171	CHRNA7-FAM7A fusion protein

P172	Cholinesterase
P173	Chitinase B
P174	Chitinase
P177	Cell division protein kinase 5
P178	Cell division protein kinase 2
P179	Cathepsin S
P180	Cathepsin K
P181	Cathepsin D
P182	Cathepsin B
P185	Casein kinase II subunit alpha
P189	Carbonic anhydrase 2
P190	Carbonic anhydrase 1
P191	cAMP-specific 3',5'-cyclic phosphodiesterase 4D
P192	cAMP-specific 3',5'-cyclic phosphodiesterase 4B
P194	cAMP-dependent protein kinase inhibitor alpha
P195	cAMP-dependent protein kinase catalytic subunit alpha
P196	Calmodulin
P197	B-Raf proto-oncogene serine/threonine-protein kinase
P198	Beta-secretase 1
P199	Beta-lactamase TEM
P200	Beta-lactamase
P201	Beta-glucosidase A
P202	Beta-2 adrenergic receptor
P204	Basic fibroblast growth factor receptor 1
P205	Bacillolysin
P206	ATP-binding cassette transporter sub-family C member 8
P207	Aralkylamine dehydrogenase light chain
P211	Amine oxidase [flavin-containing] B
P212	Amine oxidase [flavin-containing] A
P213	Alpha-7 nicotinic cholinergic receptor subunit
P214	Alpha-2C adrenergic receptor
P215	Alpha-2B adrenergic receptor
P218	Alpha-1B adrenergic receptor
P219	Alpha-1A adrenergic receptor
P220	Aldose reductase
P221	Alcohol dehydrogenase 1C
P222	Alcohol dehydrogenase 1B
P223	Alcohol dehydrogenase 1A
P224	Adenosine deaminase
P225	Adenosine A2a receptor
P227	Acetylcholinesterase
P228	5-hydroxytryptamine 4 receptor
P229	5-hydroxytryptamine 3 receptor
P230	5-hydroxytryptamine 2C receptor
P231	5-hydroxytryptamine 2B receptor
P232	5-hydroxytryptamine 2A receptor
P233	5-hydroxytryptamine 1E receptor
P234	5-hydroxytryptamine 1D receptor

P235	5-hydroxytryptamine 1B receptor
P236	5-hydroxytryptamine 1A receptor
P237	3-phosphoinositide-dependent protein kinase 1
P238	(3R)-hydroxymyristoyl-acyl carrier protein dehydratase
P239	(3R)-hydroxymyristoyl-[acyl-carrier-protein] dehydratase
P240	Voltage-dependent L-type calcium channel subunit alpha-1D
P241	Nicotinic acid receptor 2
P242	Neopullulanase 1
P243	Mineralocorticoid receptor
P244	Mast/stem cell growth factor receptor
P245	Hypoxia-inducible factor 1 alpha
P246	Histamine H2 receptor
P247	Glucosylceramidase
P248	Glutamate [NMDA] receptor subunit epsilon-1
P249	Glutamate [NMDA] receptor subunit epsilon-2
P250	Epidermal growth factor receptor
P251	Epididymal-specific lipocalin-9
P253	Cell division protein kinase 6
P254	Carboxypeptidase A1
P255	Beta-3 adrenergic receptor
P256	Alpha-mannosidase 2
P257	Amiloride-sensitive sodium channel subunit alpha
P258	Acetyl-CoA carboxylase 2
P259	5-hydroxytryptamine 1F receptor

* Targets associate with cardiovascular disease
